# The A_2B_ Adenosine Receptor Modulates the Epithelial– Mesenchymal Transition through the Balance of cAMP/PKA and MAPK/ERK Pathway Activation in Human Epithelial Lung Cells

**DOI:** 10.3389/fphar.2018.00054

**Published:** 2018-01-31

**Authors:** Chiara Giacomelli, Simona Daniele, Chiara Romei, Laura Tavanti, Tommaso Neri, Ilaria Piano, Alessandro Celi, Claudia Martini, Maria L. Trincavelli

**Affiliations:** ^1^Department of Pharmacy, University of Pisa, Pisa, Italy; ^2^Department of Surgical, Medical and Molecular Pathology and Critical Care Medicine, University of Pisa, Pisa, Italy; ^3^Radiology Unit, University Hospital of Pisa, Pisa, Italy; ^4^Pneumology Unit, Cardio-Thoracic Department, University Hospital of Pisa, Pisa, Italy

**Keywords:** adenosine A_2B_ receptor, epithelial mesenchymal transition, lung, MAPK-ERK, cAMP-PKA, transforming growth factor-β1

## Abstract

The epithelial-mesenchymal transition (EMT) is a complex process in which cell phenotype switches from the epithelial to mesenchymal one. The deregulations of this process have been related with the occurrence of different diseases such as lung cancer and fibrosis. In the last decade, several efforts have been devoted in understanding the mechanisms that trigger and sustain this transition process. Adenosine is a purinergic signaling molecule that has been involved in the onset and progression of chronic lung diseases and cancer through the A_2B_ adenosine receptor subtype activation, too. However, the relationship between A_2B_AR and EMT has not been investigated, yet. Herein, the A_2B_AR characterization was carried out in human epithelial lung cells. Moreover, the effects of receptor activation on EMT were investigated in the absence and presence of transforming growth factor-beta (TGF-β1), which has been known to promote the transition. The A_2B_AR activation alone decreased and increased the expression of epithelial markers (E-cadherin) and the mesenchymal one (Vimentin, N-cadherin), respectively, nevertheless a complete EMT was not observed. Surprisingly, the receptor activation counteracted the EMT induced by TGF-β1. Several intracellular pathways regulate the EMT: high levels of cAMP and ERK1/2 phosphorylation has been demonstrated to counteract and promote the transition, respectively. The A_2B_AR stimulation was able to modulated these two pathways, cAMP/PKA and MAPK/ERK, shifting the fine balance toward activation or inhibition of EMT. In fact, using a selective PKA inhibitor, which blocks the cAMP pathway, the A_2B_AR-mediated EMT promotion were exacerbated, and conversely the selective inhibition of MAPK/ERK counteracted the receptor-induced transition. These results highlighted the A_2B_AR as one of the receptors involved in the modulation of EMT process. Nevertheless, its activation is not enough to trigger a complete transition, its ability to affect different intracellular pathways could represent a mechanism at the basis of EMT maintenance/inhibition based on the extracellular microenvironment. Despite further investigations are needed, herein for the first time the A_2B_AR has been related to the EMT process, and therefore to the different EMT-related pathologies.

## Introduction

The epithelial-mesenchymal transition (EMT) is an evolutionarily conserved biochemical process in which cells undergo conversion from an epithelial to a mesenchymal phenotype. The EMT is characterized by the loss of the epithelial cell–cell adhesion molecule CDH1 (E-cadherin), and/or a concomitant gain of mesenchymal markers such as CDH2 (N-cadherin), VIM (Vimentin), and/or αSMA (alpha-smooth muscle actin) (Nieto et al., 2016; Singh et al., 2017). EMT is a Janus-faced process due to its pivotal role in embryogenesis and wound healing and in the development of chronic pathologies, such as fibrosis and cancer (Lamouille et al., 2014).

The EMT process has been widely correlated with lung embryogenesis (Lee et al., 2006) and cancer (Sung et al., 2016; Legras et al., 2017), but it has only recently been linked to chronic human lung and airway diseases such as chronic obstructive pulmonary disease (COPD) and idiopathic pulmonary fibrosis (IPF) (Willis et al., 2006; Sohal and Walters, 2013; Jonsdottir et al., 2015; Jolly et al., 2017). In fact, it has been proposed that alveolar epithelial cells (AECs), undergoing EMT (De Maio et al., 2012; Yamaguchi et al., 2017) or partial EMT (Morbini et al., 2011), are a source of extracellular matrix-producing fibroblasts/myofibroblasts. It has been widely accepted that several soluble factors such as, growth factors [fibroblast growth factor (FGF), epidermal growth factor (EGF)] and inflammatory cytokines (transforming growth factor-beta (TGF-β1), interleukin-6 (Il-6), tumor necrosis factor-alpha (TNF-α), could trigger and promote the EMT (Nieto et al., 2016; Suarez-Carmona et al., 2017). In the lung, attention has been focused on TGF-β1, which has been found to promote AEC differentiation (Yang et al., 2014; Shi et al., 2016) and the aggressiveness of cancer cells (Sakuma, 2017).

Adenosine is a soluble factor involved in physiological processes; however, after tissue injury, its levels rise to micromolar concentration promoting anti-inflammatory action (Ohta and Sitkovsky, 2001). Recently, a correlation between the increase in adenosine levels and the EMT process has been reported (Guillén-Gómez et al., 2012; Lu and Insel, 2014; Gao et al., 2016). Adenosine binds to four specific cell membrane G-protein-coupled receptors (GPCRs) known as adenosine receptors (ARs): A_1_, A_2A_, A_2B_, and A_3_. In this context, great attention has been focused on the purine receptor involvement in the EMT process. Only a few studies have correlated the EMT and purinergic receptors P2X and P2Y, such as P2X7, P2Y2, P2Y6, and P2Y12 (review in Martínez-Ramírez et al., 2017). Furthermore, some evidence has correlated the adenosine receptor A_2A_ with the transition (Xiao et al., 2013). However, no evidence has been reported on the role of the A_2B_AR subtype in the trigger or modulation of induced-EMT.

The A_2B_AR subtype has been recently linked to cancer aggressiveness (Mittal et al., 2016; Sepúlveda et al., 2016) and fibrotic processes of the heart (Ryzhov et al., 2014; Phosri et al., 2017), kidney (Roberts et al., 2014; Wilkinson et al., 2016) and lung (Zhou et al., 2011; Karmouty-Quintana et al., 2012; Karmouty-Quintana et al., 2013). A_2B_AR couples to G*_αs_* proteins, resulting in the activation of adenylyl cyclase (AC) and an increase in intracellular cyclic AMP (cAMP) levels that subsequently activate protein kinase A (PKA) (Schulte and Fredholm, 2003; Sun and Huang, 2016). However, A_2B_AR can also coupled to the G*_q_*-PLC pathway and induces the activation of mitogen-activated protein kinase (MAPK) (Schulte and Fredholm, 2003; Sun and Huang, 2016). In fact, A_2B_AR induces the phosphorylation of ERK1/2 in human umbilical vein endothelial cells (HUVECs) (Fang and Olah, 2007), human mast cell line (MHC-1 cells) (Feoktistov et al., 1999), and human retinal endothelial cells (HRECs) (Grant et al., 2001). The cAMP role in EMT promotion/blockade is debatable. Pattabiraman et al. (2016) reported that a PKA activator could lead to a mesenchymal-epithelial transition as previously reported by Boucher et al. (2005). Furthermore, the increase in the cAMP intracellular levels, induced by forskolin or phosphodiesterase inhibitors, as well as the administration of a PKA activator counteracts the EMT induced by TGF-β1 (Zhang et al., 2006a,b; Nadella et al., 2008; Lambers et al., 2015). By contrast, other evidence has been reported regarding the positive role of cAMP and PKA in the induction of EMT (Shaikh et al., 2012). Conversely, it has been widely accepted that ERK phosphorylation is one of the mechanisms that promotes the EMT program (Singh et al., 2017). In fact, ERK activation is one of the Smad-independent events that is necessary for TGF-β-mediated EMT (Gui et al., 2012; O’Connor and Gomez, 2014).

Therefore, in the present study, the effects of A_2B_AR stimulation on EMT was investigated in human lung epithelial cells. Particularly, the role of this adenosine receptor subtype in directly modulating the epithelial-mesenchymal markers under physiological or inflammatory conditions (TGF-β1) was analyzed. Furthermore, the intracellular pathways activated by A_2B_AR, cAMP/PKA, and MAPK/ERK, involved in EMT, were explored.

## Materials and Methods

### Material and Reagents

Human type II alveolar epithelial cells (A549, American Type Culture Collection, CCL-195), were kindly provided by Dr. R. Danesi, University of Pisa, Pisa, Italy. The chemicals 2-[6-amino-3,5-dicyano-4-[4-(cyclopropylmethoxy) phenyl]pyridin-2-ylsulfanyl] acetamide (BAY 60-6583) and N-(4-acetylphenyl)-2-[4-(2,3,6,7-tetrahydro-2,6-dioxo-1,3-dipropyl-1H-purin-8-yl)phenoxy]-acetamide (MRS1706) were purchased from Tocris Bioscience (Bristol, United Kingdom). The RNeasy minikit was obtained from Qiagen. The Script cDNA synthesis kit was furnished by Bio-Rad s.r.l. Fluocycle II SYBR was from Euroclone (Milan, Italy). TGF-β1 was purchased from Sigma–Aldrich. All other reagents were obtained from standard commercial sources and were of the highest commercially available grade

### Cell Culture

Human type II alveolar epithelial cells (A549) were maintained in DMEM-F12 containing 10% FBS, 2 mM L-glutamine, 100 U/ml penicillin and 100 μg/ml streptomycin at 37°C in a humidified 5% CO^2^ atmosphere. For the different experiments, cultures of cells were maintained in serum-free DMEM for 8 h prior to stimulation with cytokines or other reagents.

### RNA Extraction and Real-Time RT-PCR Analysis

A549 cells were untreated or treated with DMSO (CTRL) or BAY 60-6583 (100 nM) in the absence or presence of TGF-β1 (10 ng/ml) and MRS 1706 (1 μM) for the indicated time. At the end of treatments, cells were collected, and total RNA was extracted using Rneasy^®^Mini Kit (Qiagen, Hilden, Germany) as previously reported (Da Pozzo et al., 2014); residual DNA was removed using RNase free DNAse set (#79254, Qiagen, Hilden, Germany). The RNA purity was checked measuring the A260/280 ratio. cDNA synthesis was performed with 400 ng of RNA using i-Script cDNA synthesis kit (BioRad, Hercules, CA, United States) following manufacturer’s instructions. Real-time RT-PCR reactions consisted of 25 μL Fluocycle^®^II SYBR^®^ (Euroclone, Milan, Italy), 1.0 μL of both 10 μM forward and reverse primers, 2.5 μL cDNA (100 ng), and 20.5 μL of H_2_O. All reactions were performed for 40 cycles using the following temperature profiles: 98°C for 30 s (initial denaturation); 55°C for 30 s (annealing); and 72°C for 3 s (extension). The primer sequences were listed in **Table 1**. When possible, the primers used were designed to span intron/exon boundaries and the β-actin was used as the housekeeping gene. The mRNA levels for each sample were normalized against β-actin mRNA levels, and the relative expression was calculated by using a Ct value. PCR specificity was determined by both the melting curve analysis and gel electrophoresis.

**Table 1:** Primers used for real-time RT-PCR.

Gene	Primer nucleotide sequences	Product size (base pairs)	Annealing temperature
ADORA1	FOR: 5′-TCCCTCTCCGGTACAAGATG -3′ REV: 5′-GCTGCTTGCGGATTAGGTAG-3′	300 bp	55°C
ADORA2A	FOR: 5′-TCTTCAGTCTCCTGGCCATC-3′ REV: 5′-TCCAACCTAGCATGGGAGTC-3′	156 bp	55°C
ADORA2B	FOR: 5′-TCCATCTTCAGCCTTCTGGC -3′ REV: 5′-AAAGGCAAGGACCCAGAGGA-3′	129 bp	55°C
ADORA3	FOR: 5′-CAGCAAAGCGTCAACTCGTGC -3′ REV: 5′-CAAACGGGAGAAGCAGAGGAAC-3′	118 bp	55°C
CDH1	FOR: 5′-AGGGGTTAAGCACAACAGCA-3′ REV: 5′-GGGGGCTTCATTCACATCCA-3′’	395 bp	55°C
Vimentin	FOR: 5′-CTCTTCCAAACTTTTCCTCCC-3′ REV: 5′-AGTTTCGTTGATAACCTGTCC-3′	134 bp	55°C
CDH2	FOR: 5′-AGGGGACCTTTTCCTCAAGA-3′ REV: 5′-CAATGTCAATGGGGTTCTCC-3′	246 bp	55°C
ZEB1	FOR: 5′-CCCTTGAAAGTGATCCAGCCA-3′ REV: 5′-AGACCCAGAGTGTGAGAAGCG-3′	354 bp	55°C
Snail	FOR: 5′-AAGATGCACATCCGAAGCCA-3′ REV: 5′-CATTCGGGAGAAGGTCCGAG-3′	237 bp	55°C
Slug	FOR: 5′-TGGTTGCTTCAAGGACACAT-3′ REV: 5′-GTTGCAGTGAGGGCAAGAA-3′	66 bp	55°C
TWIST	FOR: 5′-ACGAGCTGGACTCCAAGATG-3′ REV: 5′-CACGCCCTGTTTCTTTGAAT-3′	290 bp	55°C
β-actin	FOR: 5′-GCACTCTTCCAGCCTTCCTTCC-3′ REV: 5′-GAGCCGCCGATCCACACG-3′	254 bp	55°C

### Western Blotting Analysis

A549 cells (3.5 × 10^4^ cell/cm^2^) were maintained untreated or treated with DMSO (CTRL) or BAY 60-6583 (100 nM) in the absence or presence of TGF-β1 (10 ng/ml) and MRS 1706 (1 μM) for 48 h. At the end 200 μl RIPA buffer were added for 60 min at 4°C to lyse the cells. Fifty microgram of total proteins was diluted in Laemmli solution, resolved by SDS-PAGE (7.5%), transferred to PVDF membranes and probed overnight at 4°C with primary anti-A_1_AR (diluted 1:200, sc-28995; Santa Cruz Biotechnology), anti-A_2A_AR (diluted 1:200, sc-13937; Santa Cruz Biotechnology), anti-A_2B_AR (diluted 1:150, sc-28996; Santa Cruz Biotechnology), anti-A_3_AR (diluted 1:200, sc-13938; Santa Cruz Biotechnology), anti-E-cadherin antibody (diluted 1:200; sc-7870; Santa Cruz Biotechnology), anti-N-cadherin antibody (diluted 1:200; sc-7939; Santa Cruz Biotechnology) anti-Vimentin (diluted 1:1000, #5741; Cell Signaling Technology) or β-actin antibody (diluted 1:1000; MAB1501, Merck KGaA, Darmstadt, Germany). The primary antibody was detected using appropriate secondary antibody. The peroxidase was detected using a chemiluminescent substrate (ECL, Perkin Elmer), and the images were acquired by photographic film or by LAS4010 (GE Health Care Europe, Uppsala, Sweden). Immunoreactive bands were quantified performing a densitometric analysis with Image J Software (version 1.41; Bethesda, MD, United States).

### Immunofluorescence Analysis

A549 cells were seeded at 3.5 × 10^4^ cell/cm^2^ in chamber slide (BD Biosciences, San Jose, CA, United States). After treatment, cells were fixed in 2% paraformaldheyde in 0.1 M phosphate buffer, washed three times with PBS, rinsed, and blocked for 45 min with PBS containing 0.1% Triton-X 100 and 1% BSA. After washing, cells were incubated overnight at 4°C with mouse monoclonal anti-β-actin antibody (diluted 1:500; MAB1501, Merck KGaA, Darmstadt, Germany) or a rabbit polyclonal anti-A_2B_AR (diluted 1:100, sc-28996; Santa Cruz Biotechnology) primary antibodies diluted in PBS containing 0.03% Triton-X 100 and 1% BSA overnight at 4°C. After washing, to visualize the staining, cells were incubated with Alexa Fluor 488- and Alexa Fluor 568-labeled goat anti-mouse (1:500) or anti-rabbit (1:500) antibodies for 2 h at room temperature. Then slides were covered with Vectashield conjugated with DAPI (Vector Laboratories, Burlingame, CA, United States). Images were obtained with a Nikon Ni-E microscope, using a 20× objective with 1.45 NA and a recommended pinhole size of less than 1.0 mm, and equipped with digital camera Nikon Mod.DS-Ri2. The images were processed with ImageJ software.

### Cell Viability Assay

A549 cells were seeded at a density of 3 × 10^3^ cells/well in a black, clear bottom PerkinElmer 96-well CellCarrier^TM^ microplate (#6005550). After 24 h, the cells were starved for 8 h with non-complete medium; then, cells were treated with different concentrations of BAY 60-6583 (0.5 nM–1 μM) or TGF-β1 (5–20 ng/ml) alone or in combination for 48 or 72 h. Following the treatment period, cells were imaged with the EnSight^TM^ multimode plate reader equipped with well-imaging module, and Kaleido Data Acquisition and Analysis Software. Brightfield images were taken before and 48 or 72 h after treatments to allow for a Cell Count comparison between both time points. The cell count was determined using the pre-defined Brightfield Cell Count algorithm provided by the Kaleido software. Cell with a mean area ≥100 μm^2^ were counted. Data were normalized to the cell count at t_0_ and expressed as the number of cell per well.

### cAMP Quantification

A549 cells were seeded at a density of 3 × 10^3^ cells/well in a black, clear bottom PerkinElmer 96-well CellCarrier^TM^ microplate (#6005550). After 24 h, the cells were starved for 8 h with non-complete medium; then, cells were treated with different concentrations of BAY 60-6583 (0.1 nM–1 μM) in the absence or presence of TGF-β1 (10 ng/ml) in 100 μl of non-complete medium for 48 h. Cells were imaged with the EnSight^TM^ multimode plate reader. The desensitization experiments were performed incubating the cells with BAY 60-6583 (100 nM) in the presence or absence of TGF-β1 (10 ng/ml) for 48 h. At the end the medium was changed and cells were treated with different concentrations of BAY 60-6583 (0.1 nM–1 μM) for 15 min. Then, cells were lysed with 50 μl of 1X lysis buffer (PerkinElmer, #AL003C) supplemented with protease inhibitors and phosphatase inhibitors for 10 min with gentle shaking. Lysates were either tested immediately in AlphaLISA assays or frozen at –80°C for later testing. cAMP levels were quantified using cAMP AlphaLISA kit (PerkinElmer, #AL312) following manufacturer’s instructions. Standard curves for each AlphaLISA immunoassay were performed in the same diluent as the samples being tested (1X lysis buffer with supplements) using the recombinant standards provided in each kit. Curves were plotted with a sigmoidal concentration-response curve with variable slope. Quantitation of protein levels in cellular assays were interpolated off their respective standard curves.

### MAPK (Mitogen-Activated Phosphorylation Kinase) Assays

A549 cells were seeded at a density of 3 × 10^3^ cells/well in 96 multi-well plate. After 24 h, the cells were starved for 8 h with non-complete medium; then, cells were treated with BAY 60-6583 (100 nM) in the absence or presence of TGF-β1 (10 ng/ml) and MRS1706 (1 μM) for 5 min, 30 min, 6, 24, and 48 h. In some experiments, before incubation with BAY 60-6583 or TGF-β1, cells were pre-treated for 30 min with PD98059 (1 μM) (MEK, inhibitor). At the end of treatments, cells were fixed with 4% formaldehyde to preserve activation of specific protein modification. Levels of total and phosphorylated extracellular signal-regulated kinases (ERK1/2) were determined by ELISA assays, as previously reported (Giacomelli et al., 2015). Briefly, the cells were washed three times with wash buffer (0.1% Triton X-100 in PBS) and 100 μl of quenching buffer (1% H_2_O_2_; 0.1% sodium azide in wash buffer) was added and incubation was protracted for other 20 min. The cells were washed with PBS twice, and then 100 μl of blocking solution (1% BSA; 0.1% Triton X-100 in PBS) was added for 60 min. After blocking, cells were washed three times with wash buffer and the specific primary antibodies (anti-phospho ERK1/2, 1:500, sc-7383 Santa Cruz Biotechnology; anti-ERK1/2, 1:500, #4695 Cell Signaling Technology) were added on at 4°C. Subsequent incubation with secondary HRP-conjugated antibodies and developing solution allowed a colorimetric quantification of total and phosphorylated levels. Blanks were obtained by treating cells in the absence of the primary antibody. The relative number of cells in each well was then determined using Crystal Violet solution. The results were calculated by subtracting the mean background from the values obtained from each test condition; values were normalized to the number of cells in each well, and were expressed as the percentage of untreated cells (basal).

### Quantification of E-cad and N-cad Proteins

A549 cells were seeded at a density of 3 × 10^3^ cells/well in a black, clear bottom PerkinElmer 96-well CellCarrier^TM^ microplate (#6005550). After 24 h, the cells were starved for 8 h with non-complete medium; then, cells were treated with different concentrations of BAY 60-6583 (0.1 nM–1 μM) in the absence or presence of TGF-β1 (10 ng/ml) in 100 μl of non-complete medium for 48 h. In some experiments, before incubation with BAY 60-6583 or TGF-β1, cells were pre-treated for 30 min with PD98059 (1 μM, MEK, inhibitor), H89 (100 nM, PKA inhibitor) or 8-Br-cAMP (100 nM–1 μM, cAMP analog & PKA activator). Then, cells were imaged with the EnSight^TM^ multimode plate reader. After imaging, cells were lysed with 50 μl of 1X lysis buffer (PerkinElmer, #AL003C) supplemented with protease inhibitors and phosphatase inhibitors for 10 min with gentle shaking. E-cad were quantified using E-cadherin AlphaLISA kit (PerkinElmer, #AL370), and N-cad were quantified using N-cadherin AlphaLISA kit (PerkinElmer, #AL379) following the manufacturer’s instructions. Standard curves for each AlphaLISA immunoassay were performed in the same diluents as the samples being tested (1X lysis buffer with supplements), using the recombinant standards provided in each kit. Curves were plotted with a sigmoidal concentration-response curve with variable slope. Quantitation of protein levels in cellular assays were interpolated off their respective standard curves.

### Statistical Analysis

The Graph-Pad Prism program (GraphPad Software Inc., San Diego, CA, United States) was used for data analysis and graphic presentation. All data are the mean ± SEM of at least three different experiments. Statistical analysis was performed by one-way analysis of variance (ANOVA) with Dunnett *post hoc* analysis to compare the data to the control, or two-way ANOVA with Bonferroni correction and two-sided tests for multiple comparisons. EC_50_ values were reported as mean of the values obtained in at least three independent experiments performed in duplicate ± SEM. P ≤ 0.05 was considered statistically significant.

## Results

### Adenosine Receptor Expression in Human Epithelial Lung Cells and Its Modulation by TGF-β1

The A549 human alveolar epithelial cells have been widely used to study the fibrotic process in the lung and related EMT mechanism (Kim et al., 2007; Ji et al., 2016). Furthermore, these cells were maintained in serum-free medium to increase the epithelial phenotype (Dong et al., 2014).

**FIGURE 1:**
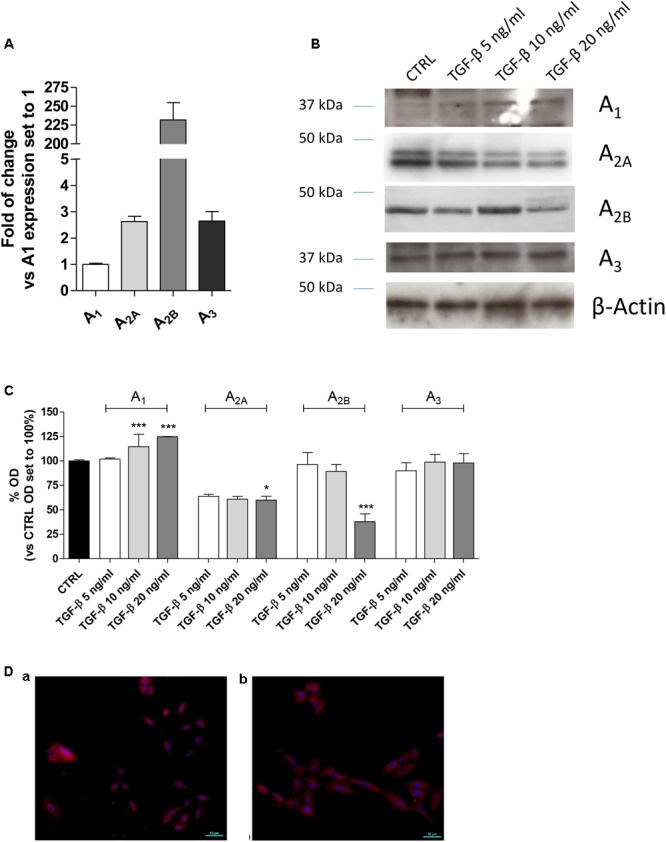
Expression of ARs in A549 cells and their modulation in the presence of TGF-β1. **(A)** A549 cells were maintained in serum-free medium for 48 h. Next, real-time RT-PCR analysis of A_1_, A_2A_, A_2B_, and A_3_ adenosine receptors was performed. The data were expressed as the fold change vs. A_1_AR expression, which was set to 1 and are the mean values ± SEM of three different experiments. **(B,C)** A549 cells were treated with different concentrations of TGF-β1 for 48 h, and the levels of AR subtypes were evaluated by Western blotting. One representative Western blot is presented **(C)**. The bar graph **(D)** shows the densitometric analysis of the Western blot performed using the ImageJ program. Cells were maintained in serum-free medium for 48 h in the absence (a) or presence (b) of TGF-β1. Next, cells were fixed and stained with anti-β actin and visualized with goat anti-rabbit Alexa Fluor 568 (red). Nuclei were counterstained with DAPI (blue). The data are presented as the means of three different experiments. The significance of the differences was determined by one-way ANOVA, followed by Dunnett’s *post hoc* test: ^∗^*P* ≤ 0.05, ^∗∗∗^*P* ≤ 0.001 vs. the CTRL.

First, the expression of the AR subtypes in A549 cells was evaluated after incubation in serum-free medium for 48 h (**Figure 1A**). All the ARs were expressed under this condition, and the A_2B_AR subtype was the most expressed with a fold change of approximately 200 (**Figure 1A**).

TGF-β1 has been reported to affect the expression of several proteins (Zhang, 2017); thus, the effects of cytokine treatment on AR expression were evaluated (**Figures 1B–D**). Challenging the A549 cells for 48 h with increasing concentrations of TGF-β1 (5–20 ng/ml) modified the expression of the different adenosine subtypes. Particularly, A_1_AR expression was significantly increased when a high concentration of TGF-β1 was used; conversely, A_2A_ and A_3_ receptor expression was slightly decreased when increased concentration of the cytokine was applied. Regarding A_2B_AR expression, low concentrations of TGF-β1 (5 or 10 ng/ml) were not sufficient to modify the receptor expression; by contrast, a higher concentration of the cytokine significantly decreased its expression. In this light, further experiments were performed using a maximum concentration of the cytokine of 10 ng/ml for 48 h to minimize the change in the receptor expression.

### TGF-β1 Effects on A_2B_AR Functionality and Lung Cell Growth

**FIGURE 2:**
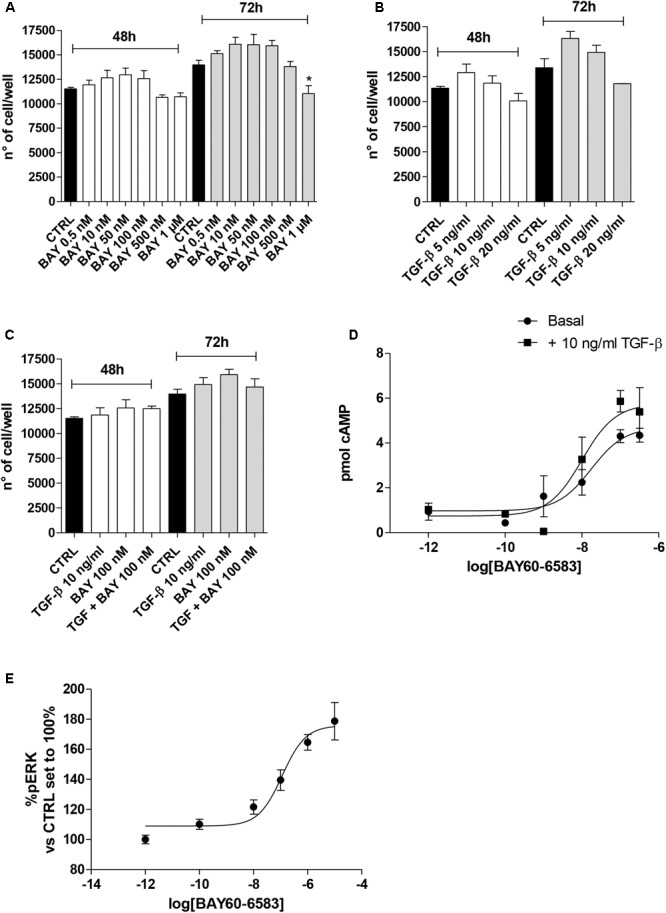
Effects of BAY 60-6583 and TGF-β1 on cell proliferation and cAMP accumulation. A549 cells were treated in serum-free medium with different concentrations of BAY 60-6583 (0.5 nM–1 μM) and TGF-β1 (5–20 ng/ml), alone or in combination, for 48 h **(A–C)**. At the end of the treatments, the cell numbers were counted. The data were expressed as cell number per well, and they were presented as the mean values ± SEM of three independent experiments, each performed in duplicate. The significance of the differences was determined by one-way ANOVA, followed by Dunnett’s *post hoc* test: ^∗^*P* ≤ 0.05 vs. the CTRL. **(D)** A549 cells were maintained in the absence or presence of TGF-β1 (10 ng/ml) for 48 h. Next, cells were challenged with increasing concentrations of BAY 60-6583, and cAMP production was quantified. **(E)** A549 cells were treated with different concentrations of BAY 60-6583 for 5 min and the levels of pERK were quantified. The data were expressed as pmol of cAMP and are the mean values ± SEM of three independent experiments, each performed in duplicate.

A_2B_AR stimulation or blockade has been correlated to different levels of cell proliferation depending on the cell type and culture condition (Phosri et al., 2017; Zhou et al., 2017). Thus, the effects of A_2B_AR stimulation in the absence or presence of TGF-β1 were evaluated in the lung cells (**Figure 2**). The selective agonist BAY 60-6583 was used to stimulate selectively the A_2B_AR subtype. The compound could slightly increase the cell proliferation when used at low concentration (50–100 nM) for 48 or 72 h treatment (**Figure 2A**), conversely, it produced a decrease in proliferation when used at a high concentration (1 μM), with a significant effect after 72 h of treatment (13970 ± 488 n° of cells CTRL, 11038 ± 816 n° of cells BAY; *P* ≤ 0.05). TGF-β1 alone presented a hormetic concentration-response course: the lower concentration possessed positive effects on cell proliferation that are lost at higher concentrations (**Figure 2B**). The combined treatment with BAY 60-6583 and TGF-β1 for 48 h did not significantly affect the cell proliferation (**Figure 2C**). These data confirmed the diverse role of A_2B_AR in the cell proliferative mechanism. Based on such results, further experiments were then performed using BAY 60-6583 at a concentration (100 nM) not affecting significantly the A549 cell proliferation.

Next, the effect of cytokine treatment on A_2B_AR functionality was evaluated by measuring the agonist-mediated cAMP production (**Figure 2D**). The potency of BAY 60-6583 was in the nanomolar range (EC_50_ = 17.2 ± 2.5 nM), as derived by agonist concentration-response curves, it is a value comparable to that described for the same agonist in transfected cells (**Figure 2D**) (Trincavelli et al., 2014). The presence of TGF-β1 did not significantly affect the agonist potency (EC_50_ = 10.0 ± 1.8 nM), even if the maximal effect of BAY in the production of cAMP was slightly increased (*E*_max_ = 4.3 pmol – TGF-β1; *E*_max_ = 5.8 pmol + TGF-β1, *P* = 0.0565) even if it was not statistically significant. These results are in accordance with the effect of other cytokines on A_2B_AR functionality (Daniele et al., 2017); in fact, TNF-α has been demonstrated to increase A_2B_AR coupling to the G*_s_* protein (Daniele et al., 2017).

In parallel, the BAY-induced ERK phosphorylation was evaluated in order to further assess the A_2B_AR functionality (**Figure 2E**). The agonist was able to produce a concentration-dependent increase of pERK with a potency in the sub-micromolar range of (EC_50_ = 114.8 ± 24.5 nM).

### Effects of A_2B_AR Stimulation on EMT Markers in the Absence or Presence of TGF-β1

**FIGURE 3:**
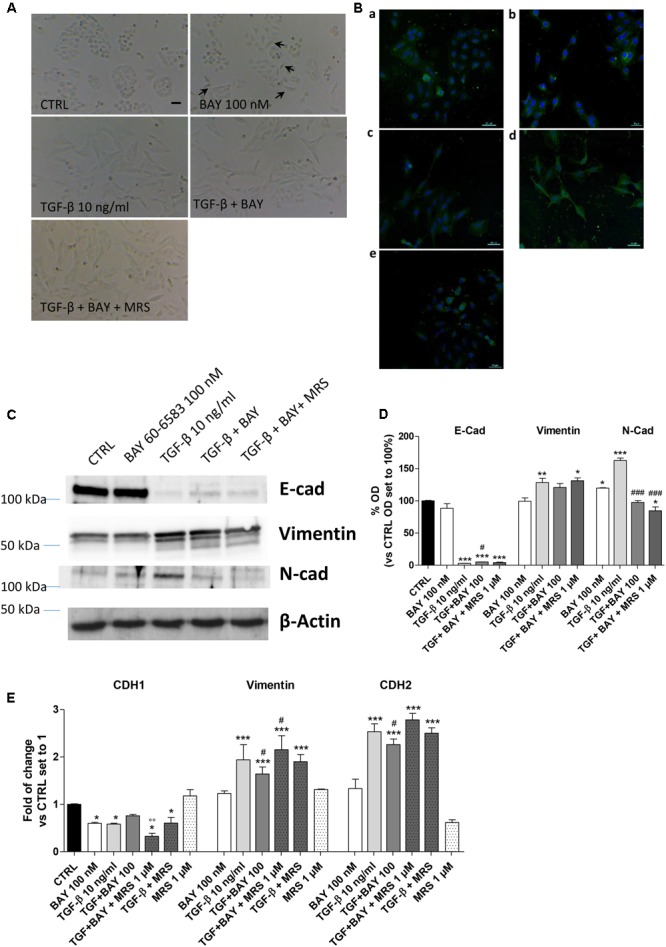
A_2B_AR stimulation affects EMT markers and decreases TGF-β1-induced EMT. A549 cells were treated with BAY 60-6583 (100 nM) in the absence or presence of TGF-β1 (10 ng/ml) for 48 h. When indicated, the A_2B_AR inverse agonist, MRS 1706 (1 μM), was applied. **(A)** At the end of the incubation times, representative images were taken. Scale bar = 100 μm. **(B)** Cells treated as above were fixed and stained with anti-β actin and visualized with goat anti-mouse AlexaFluo 488 (green). Nuclei were counterstained with DAPI (blue). **(C,D)** A549 cells were treated as described above, and the levels of the EMT markers E-cad, Vimentin and N-cad were evaluated by Western blotting. One representative Western Blot is presented **(C)**. The bar graph **(D)** shows the densitometric analysis of the Western blot performed using the ImageJ program. The data are presented as the means of three different experiments. **(E)** Real-time RT-PCR analysis of the same EMT markers were performed. The data were expressed as the fold change vs. the CTRL levels, which were set to 1 and are the mean values ± SEM of three different experiments each performed in duplicate. The significance of the differences was determined by one-way ANOVA, followed by Dunnett’s *post hoc* test or two-way ANOVA with Bonferroni correction and two-sided tests for multiple comparisons. ^∗^*P* ≤ 0.05, ^∗∗^*P* ≤ 0.01, ^∗∗∗^*P* ≤ 0.001 vs. the CTRL; ^#^*P* ≤ 0.05, ^###^*P* ≤ 0.001 vs. TGF-β1 alone; ^∘∘^*P* ≤ 0.01 vs. TGF-β1 + BAY.

Typically, the EMT process is triggered and maintained by the action of different cytokines and extracellular stimuli that, through the activation of specific intracellular pathways, modify the expression of several proteins (Nieto et al., 2016). A549 cells were treated for 48 h with the A_2B_AR agonist alone or in the presence of TGF-β1 (10 ng/ml), which has been reported to induce EMT (Kawata et al., 2012). First, a morphological analysis of the cells was performed (**Figures 3A,B**). TGF-β1 treatment induced a change in cell morphology: most of the A549 cells, which normally display an oval shape, showed an elongated shape with a fibroblast-like appearance. Surprisingly, cells challenged with the A_2B_AR agonist lead to a partial morphological change, with only some cells presenting an elongated shape, suggesting the induction of partial EMT.

The morphological changes, which are characteristic of cells undergoing EMT, are accompanied by a shift in the expression of epithelial genes to a mesenchymal gene repertoire (Nieto et al., 2016). Accordingly, challenging cells with TGF-β1 modified the expression of EMT markers, leading to a significant increase in the expression of the mesenchymal markers, Vimentin and N-cadherin (128.8 ± 6.5, *P* ≤ 0.01, and 162.7 ± 3.8% *P* ≤ 0.001 vs. CTRL, respectively; **Figures 3C,D**) and a concomitant decrease in the expression of the epithelial marker E-cadherin (6.7 ± 2.5% *P* ≤ 0.001 vs. CTRL; **Figures 3C,D**). The data were confirmed at the gene expression level by real-time RT-PCR analysis of E-cadherin (CDH1), Vimentin, and N-cadherin (CHD2) expression (**Figure 3E**).

A_2B_AR stimulation was able to slightly decrease the expression of E-cad protein (**Figures 3C,D**) and increase the expression of a mesenchymal marker (N-cad, 119.8 ± 0.6% vs. CTRL; *P* ≤ 0.05). These effects are in accordance with the effects elicited by BAY on epithelial/mesenchymal gene transcription. Conversely, A_2B_AR activation in the presence of TGF-β1 produced an opposite effect, significantly counteracting the induced EMT (**Figures 3C–E**). These effects were almost completely counteracted by concomitant treatment with the A_2B_AR selective inverse agonist MRS 1706 (1 μM), demonstrating that they were mediated specifically by A_2B_AR. The treatment with MRS 1706 alone was not able to significantly modify the expression of mesenchymal and epithelial markers in the absence or presence of TGF-β1 demonstrating that the adenosine in the medium was not sufficient to induce a basal A_2B_AR activation.

### Effects of A_2B_AR Modulation on EMT Major Transcription Factors (EMT-TFs)

**FIGURE 4:**
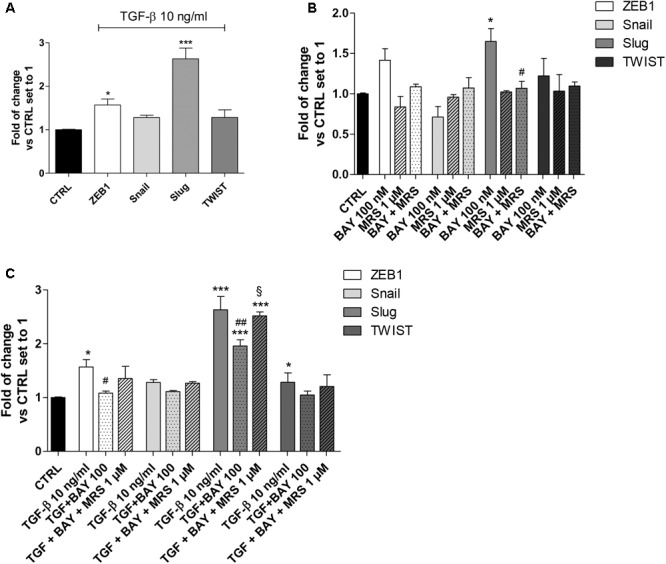
A_2B_AR activation of EMT transcription factors. **(A)** A549 cells were treated with TGF-β1 (10 ng/ml) for 48 h. At the end of the incubation, real-time RT-PCR analysis of the transcription factors that act as master regulators of EMT (ZEB1, Snail, Slug, TWIST) was performed. **(B,C)** A549 cells were treated with BAY 60-6583 (100 nM) in the absence or presence of TGF-β1 (10 ng/ml) for 48 h. When indicated, the A_2B_AR inverse agonist MRS 1706 (1 μM) was applied. At the end of the incubation, real-time RT-PCR analysis of the transcription factors that act as master regulators of EMT (ZEB1, Snail, Slug, TWIST) was performed. The data were expressed as the fold change vs. the CTRL levels, which were set to 1 and are the mean values ± SEM of three different experiments, each performed in duplicate. The significance of the differences was determined by one-way ANOVA, followed by Bonferroni’s *post hoc* test or two-way ANOVA with Bonferroni correction and two-sided tests for multiple comparisons. ^∗^*P* ≤ 0.05, ^∗∗∗^*P* ≤ 0.001 vs. the CTRL; ^#^*P* ≤ 0.05, ^##^*P* ≤ 0.01 vs. TGF-β1 alone; ^§^ *P* ≤ 0.05 vs. the TGF-β1 + BAY.

The modulation of classical EMT centers on the transcriptional control of different transcription factors: Snail (SNAI1), Slug (SNAI2), ZEB1 and TWIST (Nieto et al., 2016). To gain insight into the mechanisms by which A_2B_AR affects E-cadherin deficiency alone and in TGF-β1-induced A549 cells, the expression levels of the EMT-TFs were examined using real-time RT-qPCR (**Figure 4**).

A549 cells expressed Snail and Slug at low levels (Supplementary Figure 1), in accordance with the high expression of E-cad (**Figure 2**). Treatment with TGF-β1 for 48 h changed the expression pattern of these genes (**Figure 4A**), with a significant increase in ZEB1 (1.57 ± 0.13-fold; *P* ≤ 0.05), Slug (2.63 ± 0.25-fold; *P* ≤ 0.001), in accordance with the literature data (Ji et al., 2016). Next, the effects of A_2B_AR stimulation (BAY 60-6583) or blockade (MRS 1706) were evaluated (**Figure 4B**). The MRS alone could not modify the expression of EMT-TFs, highlighting that the endogenous activation of the receptor was not sufficient to modify the related-EMT gene expression. Conversely, A_2B_AR activation modified the gene repertoire, producing a significant increase in the Slug expression (1.65 ± 0.16-fold; *P* ≤ 0.05). These data are in accordance with the effects of A_2B_AR activation on epithelial/mesenchymal markers (**Figure 3**).

Next, the role of A_2B_AR stimulation on EMT-TF was evaluated in the presence of TGF-β1 (**Figure 4C**). As expected, the A_2B_AR activation counteracted the effects of cytokine treatment, with a significant decrease in ZEB1 and Slug expression. When the A_2B_AR inverse agonist was applied, the TGF-β1-induced decrease in gene expression was completely counteracted, demonstrating that these effects were mediated by A_2B_AR.

### The Balance of cAMP Production and MAPK/ERK Activation Orchestrates the A_2B_AR Effects on EMT

**FIGURE 5:**
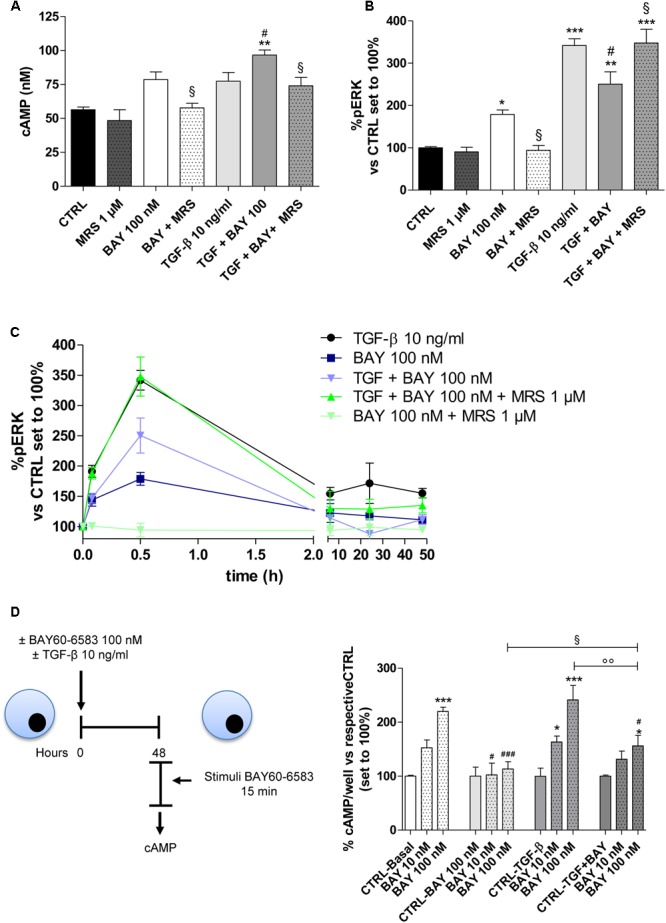
Effects of BAY 60-6583 and TGF-β1 on cAMP accumulation and ERK 1/2 phosphorylation. **(A)** A549 cells were treated with BAY 60-6583 (100 nM) in the absence or presence of TGF-β1 (10 ng/ml) for 48 h. When indicated, the A_2B_AR inverse agonist, MRS 1706 (1 μM), was applied. At the end of the treatment, the cAMP production was quantified. The data were expressed as cAMP concentrations and were presented as mean values ± SEM of three independent experiments, each performed in duplicate. **(B,C)** Cells treated as above for different time (30 min-48 h), were fixed, and ERK 1/2 phosphorylation was measured by immunoenzymatic assay. **(B)** ERK 1/2 phosphorylation after 30 min of treatment. **(C)** Time-course of ERK 1/2 phosphorylation in A549 cells. The data were expressed as the percentage versus untreated cells (CTRL) set to 100% ± SEM of at least three different experiments performed in duplicate. ^∗^*P* ≤ 0.05, ^∗∗^*P* ≤ 0.01, ^∗∗∗^*P* ≤ 0.001 vs. the CTRL; ^#^*P* ≤ 0.05 vs. TGF-β1 alone; ^§^ *P* ≤ 0.05 vs. the TGF-β1 + BAY. **(D)** Cells were treated with BAY 60-6583 (100 nM) in the absence or presence of TGF-β1 (10 ng/ml) for 48 h. After extensive washing, cells were treated for 15 min with different concentrations of BAY60-6583. Next, intracellular cAMP levels were evaluated. The data were expressed as the percentage versus untreated cells (CTRL-Basal) set to 100% ± SEM of at least three different experiments performed in duplicate. ^∗^*P* ≤ 0.05, ^∗∗∗^*P* ≤ 0.001 vs. the CTRL-Basal; ^#^*P* ≤ 0.05, ^###^*P* ≤ 0.001 vs. respective basal condition. The significance of the differences was determined by one-way ANOVA, followed by Dunnett’s *post hoc* test or two-way ANOVA with Bonferroni correction and two-sided tests for multiple comparisons.

A_2B_AR couples to G*_αs_* proteins, leading to an increase in intracellular cAMP, and to the G*_q_*-PLC pathway, which induces the activation of the MAPK/ERK pathway (Schulte and Fredholm, 2003; Sun and Huang, 2016). Thus, activation of these two intracellular signaling pathways were investigated following A_2B_AR stimulation, in the absence or presence of TGF-β1 (**Figure 5**). MRS 1706 alone did not affect the activation of the two pathways, demonstrating that the adenosine levels in our cellular model were not sufficient to activate the receptor. BAY was able to increase the intracellular cAMP concentration (**Figure 5A**), and significantly enhance ERK1/2 phosphorylation (178.9 ± 10.5% vs. CTRL, *P* ≤ 0.05; **Figure 5B**), producing transient activation of these kinases (**Figure 5C**). The effects evoked by A_2B_AR stimulation were almost completely reversed by the A_2B_AR inverse agonist MRS1706, demonstrating the specific involvement of the A_2B_AR subtype. As expected, TGF-β1 did not produce a significant increase in the cAMP levels (**Figure 5A**); conversely, it was able to significantly increase ERK1/2 phosphorylation (341.7 ± 16.2% vs. CTRL, *P* ≤ 0.001; **Figure 5B**). Both BAY and TGF-β1 induced transient activation of ERK1/2 but with different kinetics; whereas ERK phosphorylation induced by BAY peaked within 30 min and then returned to the basal value within 6 h, TGF-β1 caused a more sustained ERK activation up to 48 h of cell treatment (48 h, 155.2 ± 7.8%, TGF-β1; 85.3 ± 8.8%, BAY; *P* ≤ 0.05; **Figure 5C**). These data highlighted a difference in the final reorganization of the intracellular pathways activated by the cytokine and the receptor.

When A_2B_AR was activated in the presence of TGF-β1, the receptor activation produced a significant increase in the intracellular cAMP concentration (56.4 ± 1.9 nM, CTRL; 96.6 ± 5.3 nM, TGF-β1 + BAY *P* ≤ 0.01; **Figure 5A**). This effect was in accordance with the slight increase in A_2B_AR functionality that was noticed in the presence of the cytokine (**Figure 2D**). Regarding pERK1/2, the co-treatment led to a modest decrease in phosphorylation. The BAY activities on cAMP and pERK1/2 were most completely counteracted by cell pre-incubation with the A_2B_AR inverse agonist MRS1706. Finally, TGF-β1 and BAY did not affect the amount of total ERK (Supplementary Figure 2).

To elucidate if the different activation of cAMP and ERK pathway in the presence of the cytokine, could be related to A_2B_AR desensitization, the functionality of the receptor was evaluated after 48 h of cell treatment in the absence or presence of BAY and TGF-β1 alone or in combination (**Figure 5D**). In the basal condition (48 h without treatment), A_2B_AR maintained its functional response. The cell treatment with BAY (100 nM) for 48 h induced the desensitization of the A_2B_ receptor (219.9 ± 7.7% in Basal condition; 113.3 ± 13.6% in BAY pre-treated cells; *P* ≤ 0.001). Cytokine alone was not able to significantly interfere on the receptor desensitization. Interestingly, when cells were treated with BAY in the presence of TGF-β1 for 48 h, the desensitization of the receptor was partially impaired (164.2 ± 8.7%; *P* ≤ 0.01).

### cAMP/PKA Regulation of EMT Markers in Human Epithelial Cells

**FIGURE 6:**
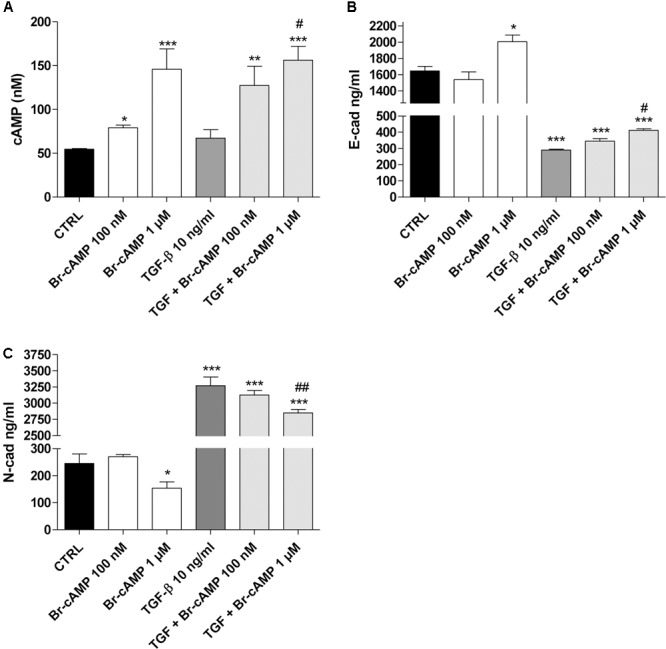
Effect of the cAMP pathway on E-cadherin and N-cadherin expression. A549 cells were treated with Br-cAMP (100 nm–1 μM) in the absence or presence of TGF-β1 (10 ng/ml) for 48 h. **(A)** At the end of the treatment, cAMP production was quantified. The data were expressed as cAMP concentrations and are presented as mean values ± SEM of three independent experiments, each performed in duplicate. Cells treated as above were lysed, and the expression of E-cadherin **(B)** or N-cadherin **(C)** was quantified using AlphaLISA kits. The data were expressed as E-cadherin and N-cadherin concentrations (ng/ml) and are presented as mean values ± SEM of three independent experiments, each performed in duplicate. The significance of the differences was determined by two-way ANOVA with Bonferroni correction and two-sided tests for multiple comparisons. ^∗^*P* ≤ 0.05, ^∗∗^*P* ≤ 0.01, ^∗∗∗^*P* ≤ 0.001 vs. the CTRL; ^#^*P* ≤ 0.05, ^##^*P* ≤ 0.01, vs. TGF-β1 alone.

The role of the cAMP pathway in EMT is controversial, depending on the cell type, the nature of the EMT inducers and maximum levels of the intracellular cAMP obtained (Weng et al., 2015). Thus, the effects of different levels of cAMP on EMT markers in our cellular model were investigated using the cAMP analog Br-cAMP (8-bromoadenosine 3′,5′-cyclic monophosphate) as the PKA activator (**Figure 6**). Br-cAMP (100 nm–1 μM) induced a concentration-dependent increase in the intracellular cAMP levels (**Figure 6A**). Surprisingly, different concentrations of the activator caused opposite effects on E-cad (**Figure 6B**) and N-cad (**Figure 6C**) expression. A high Br-cAMP concentration increased E-cad and decreased N-cad expression. A low Br-cAMP concentration (100 nM) was sufficient to determine a modest decrease of E-cad (1646 ± 56 ng/ml, CTRL; 1540 ± 93 ng/ml, Br-cAMP; **Figure 6B**) and an increase of N-cad (246 ± 35 ng/ml, CTRL; 270 ± 8 ng/ml, Br-cAMP; **Figure 6C**) even if these variations were not statistically significant. When TGF-β1 and BAY were used simultaneously, the activator produced higher cAMP levels and a concentration-dependent increase in E-cad and decrease in N-cad.

These data demonstrate that, in our cellular model, when the cAMP was increased up to high concentrations, it could negatively affect the EMT process. Conversely, when cAMP levels remained low, it seems to mediate the opposite effects, promoting a slight decrease in epithelial markers and an increase in the mesenchymal markers. This controversial nature of the cAMP analogue on the EMT process is in accordance with the recent data reported by Zuccarini et al. (2017).

### The cAMP/PKA-MAPK/ERK Balance in the A_2B_AR Modulation of EMT Markers and EMT-TFs

**FIGURE 7:**
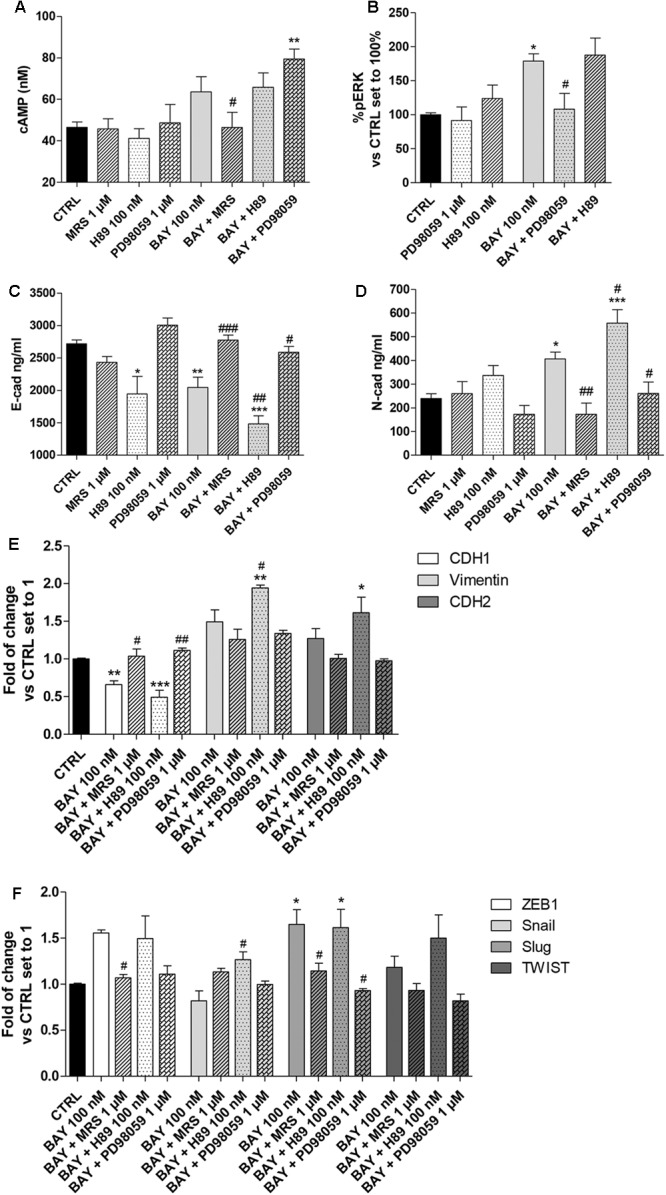
Effect of cAMP and ERK1/2 phosphorylation blockade on A_2B_AR-mediated changes in EMT markers. A549 cells were treated with BAY 60-6583 (100 nM) in the absence or presence of MRS 1706 (1 μM), PKA inhibitor H89 (100 nM) or MEK1/2 inhibitor PD98059 (1 μM) for 48 h. When indicated, the antagonist and inhibitor were applied 30 min before treatment with BAY 60-6583. **(A)** At the end of the treatment, cAMP production was quantified. The data were expressed as cAMP concentrations and are the mean values ± SEM of three independent experiments, each performed in duplicate. **(B)** Cells were treated as above*(and ERK 1/2 phosphorylation was evaluated after 30 min of treatment. The data were expressed as the percentage versus untreated cells (CTRL) set to 100%±SEM of at least three different experiments performed in duplicate. Cells treated as above were lysed, and the expression of E-cadherin **(C)** or N-cadherin **(D)** were quantified using AlphaLISA kits. The data were expressed as E-cadherin and N-cadherin concentrations (ng/ml) and are the mean values±SEM of three independent experiments, each performed in duplicate. The significance of the differences was determined by one-way ANOVA, followed by Dunnett’s *post hoc* test or two-way ANOVA with Bonferroni correction and two-sided tests for multiple comparisons. ^∗^*P* ≤ 0.05, ^∗∗^*P* ≤ 0.01, ^∗∗∗^*P* ≤ 0.001 vs. the CTRL; ^#^*P* ≤ 0.05, ^##^*P* ≤ 0.01, ^###^*P* ≤ 0.001 vs. BAY alone. **(E,F)** Cells were treated as above and real-time RT-PCR analysis of EMT markers (E-cad, N-cad and Vimentin) **(E)**, and EMT transcription factors (ZEB1, Snail, Slug, TWIST) **(F)** was performed. The data were expressed as the fold change vs. the CTRL levels, which were set to 1 and are the mean values±SEM of three different experiments each performed in duplicate. The significance of the differences was determined by one-way ANOVA, followed by Dunnett’s *post hoc* test or two-way ANOVA with Bonferroni correction and two-sided tests for multiple comparisons. ^∗^*P* ≤ 0.05, ^∗∗^*P* ≤ 0.01, ^∗∗∗^*P* ≤ 0.001 vs. the CTRL; ^#^*P* ≤ 0.05, ^##^*P* ≤ 0.01 vs. BAY alone.)*

Nevertheless, high levels of cAMP counteract EMT, and ERK phosphorylation mediated by MAPK activation has been demonstrated to promote EMT (Singh et al., 2017). Thus, the ability of A_2B_AR to modify the amounts of both pERK and of cAMP could be a possible mechanism by which the receptor agonist affects epithelial/mesenchymal markers. In this respect, the involvement of A_2B_AR in the regulation of EMT markers and transcription factors was investigated in the presence of PKA inhibitor (H89, 100 nM) and a mitogen-activated protein kinase inhibitor (PD98059, 1 μM) (**Figure 7**, Supplementary Figure 3). The BAY-mediated increase in the intracellular cAMP levels was not affected by H89 treatment (**Figure 7A**); conversely, PD98059 was able to block BAY-mediated ERK phosphorylation (**Figure 7B**). In this respect, the inhibitors were used to unveil the involvement of the two signaling pathways in A_2B_AR mediated triggering of EMT.

BAY decreased the protein density of E-cad and increased that of the N-cad one; these effects were lost in the presence of the inverse agonist MRS 1706, in accordance with the abovementioned data (**Figure 3**). The ability of BAY to decrease the expression of E-cad was augmented by pretreatment with the PKA inhibitor (2718 ± 58 ng/ml, CTRL; 2045 ± 157 ng/ml, BAY; *P* ≤ 0.01; 1483 ± 122 ng/ml, BAY + H89; *P* ≤ 0.001 vs. CTRL, *P* ≤ 0.01 vs BAY, **Figure 7C**). Similarly, A_2B_AR mediated increase in N-cad was enhanced when the cAMP/PKA axis was abrogated (239 ± 20 ng/ml, CTRL; 406 ± 29 ng/ml, BAY; *P* ≤ 0.05; 558 ± 72 ng/ml, BAY + H89; *P* ≤ 0.001 vs. CTRL, *P* ≤ 0.05 vs BAY; **Figure 7D**). These effects highlighted that cAMP, through PKA activation, could partially antagonize A_2B_AR-mediated EMT induction. When the MEK inhibitor was used, the changes induced by A_2B_AR stimulation were almost completely counteracted, restoring the basal conditions (E-cad, 2586 ± 90 ng/ml, BAY + PD98059; *P* ≤ 0.05 vs BAY; N-cad, 260 ± 49 ng/ml, BAY + PD89059; *P* ≤ 0.05 vs BAY, **Figures 7C,D**). These results are in accordance with the effects of the inhibitor treatments on A_2B_AR-mediated alteration of the expression of CDH1, CDH2 and vimentin genes (**Figure 7E**).

Finally, the modulation of the EMT-TF gene expression by BAY was evaluated in the presence of H89 and PD98059. Similarly, regarding the EMT markers, the PKA inhibitor slightly exacerbated the A_2B_AR-mediated effects; conversely, the MEK inhibitor counteracted the BAY-mediated increase in gene expression. The PD98059 activity was more pronounced on Slug expression, causing a significant decrease of gene expression (1.65 ± 0.16-fold, BAY; 1.61 ± 0.20-fold, BAY + H89; 0.93 ± 0.09-fold vs. CTRL, BAY + PD98059; *P* ≤ 0.05). These data are consistent with the reported role of ERK phosphorylation in the induction of Slug expression (Choi et al., 2007; Joannes et al., 2014).

## Discussion

**FIGURE 8:**
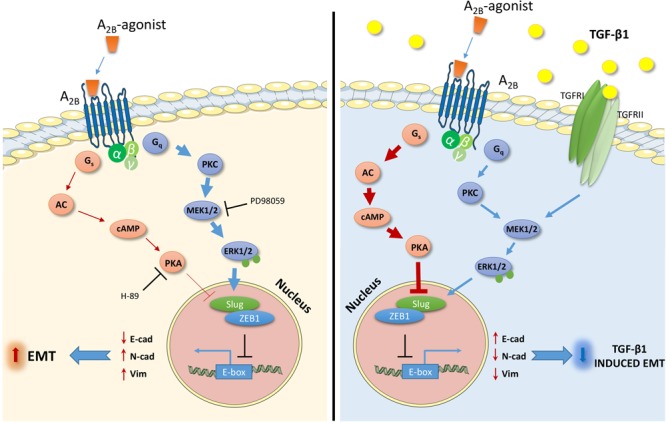
Schematic representation of the effects and the intracellular signals activated by the A_2B_AR stimulation in the absence **(left)** or presence **(right)** of TGF-β1. The A_2B_AR activates cAMP/PKA pathways and ERK1/2 phosphorylation. The extent of the activation changes in the presence of the TGF-β1 cytokine **(bold arrow)**, causing different effects on the expression of transcription factors and markers related to EMT. The receptor activation increases the EMT process **(left)**, and counteracts the TGF-β1-induced EMT **(right)**.

Adenosine receptors (ARs) have attracted great attention as possible targets to control the EMT process in pathologies such as fibrosis and cancer (Lu and Insel, 2014). However, among the AR subtypes, no data have been reported that correlate A_2B_AR activation with EMT in epithelial cells. In this respect, herein, we report for the first time the ability of A_2B_AR activation to promote EMT or contrast the effects of the extracellular inducer, TGF-β1, in human lung epithelial cells. Furthermore, the ability of the receptor activation to modulate two signaling pathways involved in EMT, cAMP/PKA, and MAPK/ERK, was demonstrated as a possible mechanism explaining the different effects mediated by A_2B_AR stimulation in different extracellular microenvironment (**Figure 8**).

Human carcinoma epithelial lung cells (A549) were maintained under serum deprivation, a condition that has been demonstrated to amplify the epithelial phenotype (Dong et al., 2014). Although all the ARs are expressed in epithelial lung cells, the A_2B_ receptor subtype was demonstrated to be the most represented, as previously reported (Roman et al., 2006). Challenging these cells with TGF-β1 produced a marked change in the phenotype through the simultaneous decrease in epithelial markers (E-cad) and increase in mesenchymal markers (N-cad). In accordance with the literature (Kawata et al., 2012), these data demonstrated the suitability of the model to study the A_2B_AR involvement in the EMT process.

A_2B_AR stimulation modified the expression of epithelial/mesenchymal markers with a low but significant amplitude. Surprisingly, the same receptor could counteract the EMT induction mediated by TGF-β1. These opposite effects elicited by A_2B_AR stimulation in lung cells reflect the well documented debated role of this receptor in the onset and progression of different pathologies. The receptor activation has been reported to protect the lungs and other tissues from inflammation and acute injury (Eckle et al., 2008; Davies et al., 2014; Hoegl et al., 2015). By contrast, other evidence has been reported concerning the A_2B_AR-mediated pro-fibrotic effects and promotion of cancer development (Karmouty-Quintana et al., 2012; Huerter et al., 2016; Sepúlveda et al., 2016). Furthermore, Zhou et al. (2011) have shown that A_2B_AR genetic removal slightly affects acute lung injury but reduces lung fibrosis, supporting a pro-fibrotic role for this receptor.

In our study, the A_2B_AR selective agonist regulated the gene transcription of different EMT-transcription factors (EMT-TFs). EMT is a complex process regulated by the orchestration of several transcription factors. Among these EMT-TFs, ZEB1, Snail and Slug (SNAI2) are the major transcription factors involved in the promotion of the EMT process in A549 cells, as previously reported (Ji et al., 2016). A_2B_AR stimulation did not affect significantly the expression of Snail and TWIST, other master genes of EMT. However, A_2B_AR activation modulate the expression of Slug and, to a lesser extent, that of ZEB1, which are commonly considered the major actors in myofibroblast differentiation and fibrogenesis (Lamouille et al., 2014). Furthermore, Slug expression has been correlated to direct repression of E-cadherin expression (Choi et al., 2007). This evidence was in accordance with the increase in E-cadherin induced by A_2B_AR stimulation.

The EMT-TFs are regulated by the activation of different intracellular pathways, which in turn regulate the balance between the epithelial and mesenchymal markers. ERK1/2, GSK3β, p38, STAT3, and cAMP are only few of all the proteins involved in the promotion/inhibition of EMT (Nieto et al., 2016). In our cellular model, activation of the cAMP/PKA axis promoted opposite effects depending on the levels of intracellular the cAMP. Low concentrations led to a slight promotion or no effects on epithelial/mesenchymal markers; conversely, high levels of cAMP could counteract EMT progression. In accordance with our results, it has been reported that cAMP/PKA activation could exert opposite effects on EMT (Zhang et al., 2006a,b; Nadella et al., 2008; Shaikh et al., 2012; Lambers et al., 2015) that could be ascribed to the different cell models, type of EMT inducer and maximum levels of intracellular cAMP obtained (Weng et al., 2015).

A_2B_AR activation increases the intracellular cAMP levels and promotes the phosphorylation of ERK 1/2 (Grant et al., 2001; Zhou et al., 2017). In this respect, we hypothesize that the controversial role of A_2B_AR in promoting EMT or reversing TGF-β1-induced EMT could be ascribed to the different balance of the intracellular pathways activated. The activation of other GPCRs coupled to different G proteins has been reported to elicit opposite effects on EMT. Zhang et al. (2016) have demonstrated that norepinephrine, which activates G*_s_* protein, induces the epithelial-mesenchymal transition in A549 cells; similarly, the P2X7 stimulation, which activates the PI3K/Akt and ERK1/2 signaling pathways, promotes the mesenchymal phenotype in epithelial renal cells (Zuccarini et al., 2017). A_2A_ARs have been reported to modify the epithelial/mesenchymal phenotype in renal cells by downregulating TGF-β1-induced EMT (Zuccarini et al., 2017) in accordance with the effects elicited by A_2B_AR activation in our cellular model when the cytokine was present. TGF-β1 activates both Smad signaling and non-Smad signaling including the ERK pathway, critically regulating EMT (Xie et al., 2004). A_2B_AR stimulation could counteract cytokine-induced EMT, reducing the transition progression. These effects were correlated with a robust increase in cAMP production and with a concomitant decrease in ERK phosphorylation. In this respect, we could speculate that the increase in the cAMP, mediated by A_2B_AR activation, could counteract the cytokine-mediated effects on EMT. This is in accordance with the ability of other G_s_ coupled receptors (i.e., A_2A_AR) or cAMP inducers (i.e., forskolin, 8-Br-cAMP) to prevent TGF-β1-mediated EMT (Zhang et al., 2006a,b; Zuccarini et al., 2017). It should also be considered that cAMP/PKA has a negative effect on TGF-β induced pERK 1/2, but only when the cytokine downstream pathways are highly expressed (Weng et al., 2015). This mechanism could explain the reduction in ERK1/2 phosphorylation in the presence of the A_2B_AR agonist.

The modulation of cAMP production and ERK phosphorylation could be affected by internalization or desensitization processes of the receptor. Several GPCRs employ a variety of signaling mechanisms to exert their functions. The production of second messengers, such as cAMP, is the results of the receptor-G protein coupling. However, other signals produced in conjunction with, or even independent of heterotrimeric G protein could be activated. It is well known that the arrestins, a family of four GPCR-binding proteins originally described for their role in the GPCR desensitization, have been found to interact with Src family kinases and to serve as scaffolds for activation of ERK1/2 and c-Jun N-terminal kinase 3 mitogen-activated protein kinases (Ferguson, 2001; Luttrell, 2005; Lee et al., 2008). Thus, the desensitization could represent a mechanism to maintain the activation of G-protein independent pathways. The activation of the A_2B_AR for long time produced the desensitization of the receptor. This may be another mechanism through which A_2B_AR mediate the ERK phosphorylation favoring the EMT traits. Conversely, the TGF-β1 was able to reduce the agonist-induced A_2B_AR desensitization, maintaining the ability of A_2B_AR to produce cAMP that negatively affected EMT process. These data could better highlight the controversial role of A_2B_AR in the EMT. In different pathological status in which adenosine raise to micromolar concentration, the extracellular microenvironment and the localization of the receptor could interfere with the levels of the activated intracellular signaling shifting the balance in favor of or against the EMT.

In our cellular model, A_2B_AR activation was able to induce partial EMT; however, it was not sufficient to promote a complete epithelial-mesenchymal transformation of the lung cells. The involvement of the cAMP/PKA and MAPK/ERK signaling pathways was demonstrated by the use of selective inhibitors. The EMT traits, in the presence of A_2B_AR agonist was enhanced by PKA inhibition and decreased by the ERK1/2 phosphorylation blockade. Furthermore, the pretreatment with a PKA inhibitor did not enhance receptor effects on TF expression. Conversely, the presence of the MEK inhibitor completely counteracted the A_2B_-mediated increase in Slug and ZEB1 gene expression, demonstrating the involvement of MAPK/ERK pathway activation in A_2B_AR-mediated EMT induction.

## Conclusion

For the first time, these results highlight the possibility that A_2B_AR is one of the numerous receptors that could be involved in EMT regulation. Although the expression of the other adenosine receptor subtypes are low, in physiological condition the involvement of all the AR in the EMT regulation could not be excluded. A_2B_AR activation is not enough to trigger a complete transition; it can affect the expression of the epithelial and mesenchymal markers promoting EMT traits. These effects are related to the ability of A_2B_AR to modify the balance between cAMP/PKA and MAPK/ERK activation. The presence of extracellular cytokines that activate the transition could change the balance and levels of these two intracellular pathways, shifting the effects exerted by the receptor stimulation on EMT trigger and maintenance. Thus, the A_2B_AR seems to act as an EMT regulator rather than a main actor in the EMT induction. Although the EMT is a complex machinery and further investigation are needed to better elucidate the effects of adenosine and the contribution of the other AR subtypes in physiopathological conditions, herein, for the first time, the A_2B_AR was shown to be related to the EMT process, highlighting its potential role as pharmacological target in EMT-related pathologies.

## Author Contributions

CG designed and performed the biological experiments. CG, SD, and MT. analyzed the data and wrote the manuscript. IP performed the immunofluorescence analysis. CR, LT, and TN contributed to the experimental work. MT, AC, and CM designed the study and played a key role as project supervisor. MT and CM coordinated the project. All the authors contributed to and approved the final manuscript.

## Conflict of Interest Statement

The authors declare that the research was conducted in the absence of any commercial or financial relationships that could be construed as a potential conflict of interest.
