# Supplementary material for: The A2B Adenosine Receptor Modulates the Epithelial– Mesenchymal Transition through the Balance of cAMP/PKA and MAPK/ERK Pathway Activation in Human Epithelial Lung Cells
Source: Front Pharmacol. 2018 Jan 31;9:54. doi: 10.3389/fphar.2018.00054 (PMC5797802; doi:10.3389/fphar.2018.00054)

Supplementary Material

The A_2B_ adenosine receptor modulates the epithelial-mesenchymal transition through the balance of cAMP/PKA and MAPK/ERK pathway activation in human epithelial lung cells.

Chiara Giacomelli^1^, Simona Daniele^1^, Chiara Romei^2,3^, Laura Tavanti^4^, Tommaso Neri^2^, Ilaria Piano^1^, Alessandro Celi^2^, Claudia Martini^1*^, Maria Letizia Trincavelli^1^

*** Correspondence:** Claudia Martini: [claudia.martini@unipi.it](mailto:claudia.martini@unipi.it)

# Supplementary Figures and Tables

## Supplementary Figures 1

**Supplementary Figure 1.** Expression of EMT-TFs in human epithelial lung cells A549. Cells were maintained in non-complete medium for 48h. At the end, the total mRNA were extracted and a real-time RT-PCR analysis was performed. The data were expressed as the ΔCT vs. the levels of the β-actin, and were the mean values ± SEM of three different experiments.

## Supplementary Figures 2

**Supplementary Figure 2.** Modulation of BAY 60-6583 and TGF-β1 on total ERK 1/2. A549 were treated for different times (0-50 h) with BAY 60-6583 (100 nM) in the absence or presence of TGF-β1 (10 ng/ml) and MRS1706 (1 µM); then, total ERK was quantified by immunoenzymatic assay. The data are expressed as the percentage versus untreated control cells (set to 100%) ± SEM of three different experiments.

## Supplementary Figures 3

**Supplementary Figure 3.** Modulation of ERK 1/2 phosphorylation. A549 cells were treated for 30 min with TGF-β1 (10 ng/ml), where indicated 30 min prior the PD98059 1 µM were added; at the end of the treatment period, the total ERK was quantified by immunoenzymatic assay. The data are expressed as the percentage versus untreated control cells (set to 100%) ± SEM of three different experiments. different experiments. The significance of the differences was determined by one-way ANOVA, followed by Bonferroni’s post hoc test. ***P ≤ 0.001 vs. the CTRL; ### P ≤ 0.001 vs. TGF-β1 alone.

**“Full-length blots relative to the cropped images showed in the main Figures”**

**Figure 1**


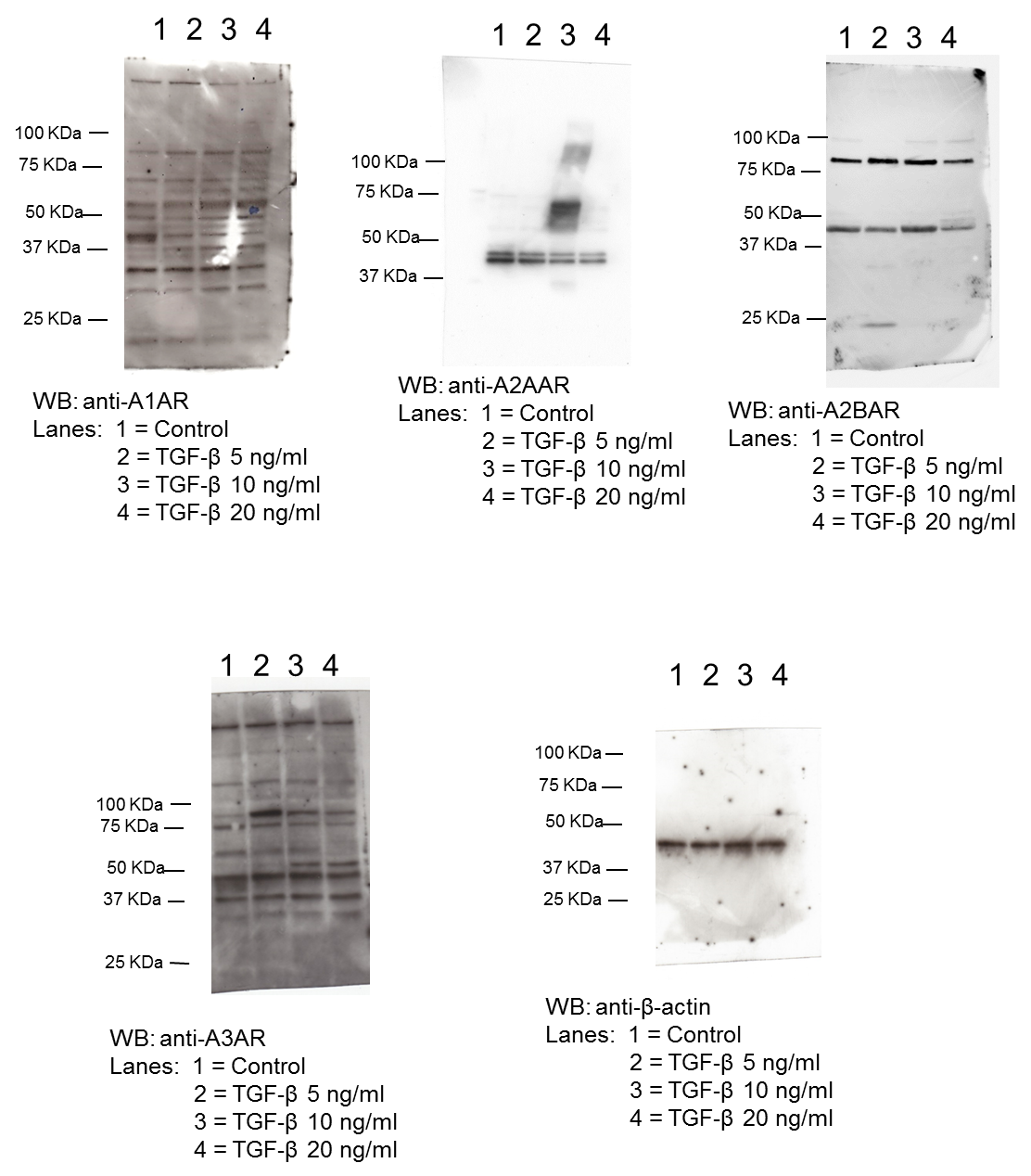


**“Full-length blots relative to the cropped images showed in the main Figures”**

**Figure 3**


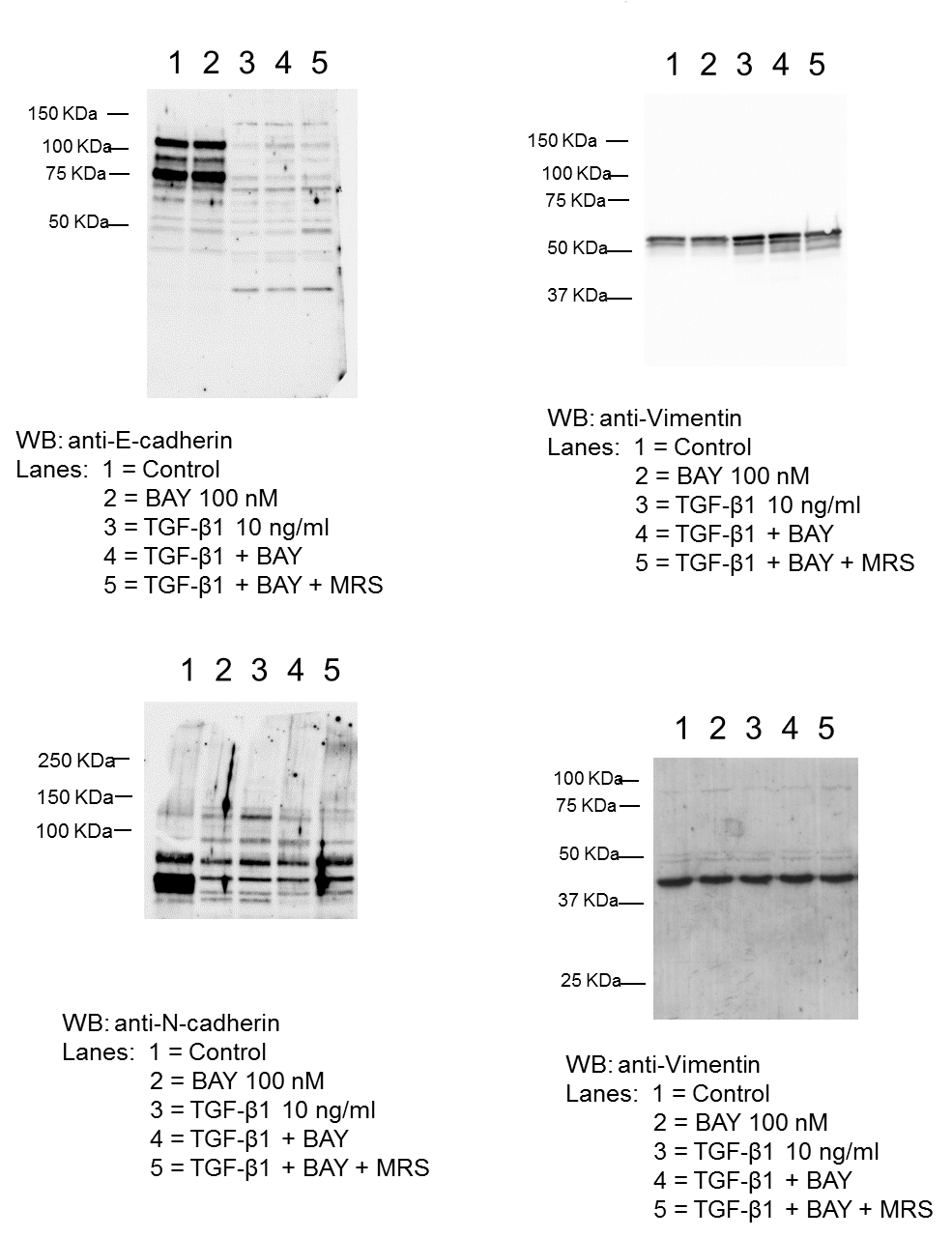

Supplement: Supplementary file 1 [file Data_Sheet_1.DOCX]
